# Does Committing to Green Goals Always Lead to Impactful Action? An Identity‐Goal Perspective

**DOI:** 10.1111/nyas.70363

**Published:** 2026-07-30

**Authors:** Katarzyna Byrka, Simona Sciara, Johannes T. Doerflinger, Peter M. Gollwitzer

**Affiliations:** ^1^ SWPS University Wroclaw Poland; ^2^ Vita‐Salute San Raffaele University Milan Italy; ^3^ University of Freiburg Freiburg Germany; ^4^ University of Konstanz Konstanz Germany; ^5^ New York University New York USA; ^6^ Zeppelin University Friedrichshafen Friedrichshafen Germany

**Keywords:** environmentalism, moral identity goals, online activism, self‐completion theory, slacktivism

## Abstract

Many people see caring for the environment as part of who they are and what they believe is morally right. Yet even when protecting nature is valued, consistently acting in environmentally friendly ways in everyday life can be difficult. In this paper, we adopt an identity‐goal perspective and discuss why people who set green goals and feel environmentally responsible may choose quick and easy actions that signal concern but have limited real impact. Committing to the identity goal of being a green person might be the best route to success. However, there may also be potential downsides. When people feel they have already done enough to see themselves as environmentally responsible, they may stop putting in further effort—a pattern often called “slacktivism.” At the same time, people who feel they have not done enough may look for quick and easy actions that make them feel better, rather than taking more effective steps that require time and effort. This is problematic because environmental protection requires sustained effortful action over time. We will therefore discuss potentially effective self‐regulation strategies that help to overcome these problems.

## 1 Introduction

Goals are known to promote goal‐directed action. Thus, committing oneself to the goal of being a moral person should enhance the expression of moral behaviors. In the same vein, given that eco‐friendliness is viewed as an expression of one's morality, committing to environmental protection should suffice to align one's actions with proenvironmental values. While this may seem intuitively obvious, the reality is more complex. For instance, it is known that having reached a set goal reduces goal striving. This becomes even more apparent when individuals pursue long‐term moral goals—such as the identity goal of becoming an environmentally responsible person. A sense of completeness and fulfillment regarding such an identity goal can actually reduce further engagement [1]. The aim of this paper is to present this goal pursuit perspective [2, 3], as a conceptual background to explain why sometimes even individuals with a strong commitment to a moral identity‐goal fail to act morally. Moreover, exemplified by the identity goal of being an eco‐friendly person, we argue that some behaviors that individuals undertake to feel complete in this identity goal are easy ways to symbolize this identity but not necessarily effective ways to protect the environment. In particular, we examine “slacktivism”—the phenomenon of supporting a social cause on social media by involving very little or no effort—as a case within the broader debate on the effectiveness and impact of individual proenvironmental behaviors. We explain how social media provide a privileged context in which individuals can feel they have achieved their moral proenvironmental identity goal without having engaged in demanding proenvironmental action. Finally, in addition to the negative consequences of slacktivism, we also discuss its potential positive aspects.

## 2 Moral Identity Goals

Morality plays a central role in lay conceptions of self‐definitions. Moral traits have been found to outweigh other mental faculties such as autobiographical memory or basic cognitive functions in shaping people's identities [4]. It stands to reason therefore that being moral is, for many people, an aspired‐to identity that they are committed to attain. Efforts and strivings people undertake to become a moral person indicate that moral identity is not merely a self‐image in terms of How moral am I? or Is it important for me to be moral? [5] but also an aspired‐to, moral identity goal [6] such as, I want to become a moral person! In other words, moral identity is shaped both by how central moral values and traits are for someone [7, 8], and by the commitment to the pursuit of a desired goal for oneself—being a moral person [9].

Understanding moral identity in terms of an aspired‐to identity goal requires a closer look at the mechanisms underlying goal striving—a process that is explicated in the symbolic self‐completion theory [10]. According to this theory, committing to an identity goal creates a readiness to acquire respective indicators (referred to as symbols) that signal goal progress. For example, a person who commits to becoming moral adopts an identity goal that involves a continuous pursuit of behaviors consistent with that identity. As soon as the person sees the need to improve, further striving is called for. And the urge to improve will arise again and again, as all kinds of hindrances and shortcomings will be encountered as the pursuit of an aspired‐to identity goal is a life‐long journey.

Identity goals can never be ultimately completed. There is always room for improvement and the person's commitment to the aspired‐to identity goal will trigger the needed actions. Whenever people are driven by an identity goal, the person's involvement with meeting this goal is thus inherently open‐ended. Moreover, renewed striving for the identity goal is often triggered by the experience of falling short—which the symbolic self‐completion theory refers to as a sense of incompleteness.

Thus, moral identity‐goal striving needs to be understood as an ongoing constructive pursuit of an aspired‐to self‐definition (e.g., being a moral person) and its maintenance. A person pursuing an aspired‐to identity goal will be faced repeatedly with evidence for and against a state of completeness. Individuals striving for identity goals will feel complete when they can point to evidence suggesting the possession of the aspired‐to identity. At this point, the urge for further goal striving is reduced. Individuals remain complete until they encounter a disruption. Losing or failing to acquire an identity‐relevant symbol leads to a sense of incompleteness associated with feeling tension [11], which motivates individuals to compensate by symbolizing the possession of the aspired‐to identity with alternative symbols. Individuals in a state of incompleteness feel an urge to turn this unpleasant state into a pleasant state of completeness by engaging in self‐symbolizing—acquiring new or pointing to already acquired indicators of possessing the aspired‐to identity. Importantly, self‐symbolizing efforts to turn an experienced incompleteness into feelings of completeness have been found to be most effective when they are noticed by others—thus acquiring social reality [12]. Relevant to this idea, a more recent meta‐analytical study confirmed that individuals who act in a moral or ethical way feel justified in engaging in behavior that is less moral at the next occasion, mostly, however, when their original moral actions were observed by others [13].

People who are committed to an identity goal can seek out all kinds of indicators of possessing the aspired‐to identity when self‐symbolizing. Such indicators serve as symbols suggesting that one is moving forward to the aspired‐to identity goal, and identity goals are commonly linked to a host of possible symbols. According to self‐completion theory, the symbols of an aspired‐to identity are interchangeable. Individuals can symbolize their identity by demonstrating identity‐relevant skills, showing behavior typical of the identity, displaying relevant physical objects that point to having been successful in the past, or merely stating intentions that one will strive for the aspired‐to identity. Most aspired‐to identities are associated with indicators of goal completeness such as relevant possessions, acquired rewards, and titles, having established relevant social connections, or demonstrating sophisticated relevant skills. Self‐symbolizing may take various forms depending on the identities involved, such as recycling waste among environmentally conscious individuals [1], choosing ecological dishes among vegans [14], or spending more time with children among committed parents [15].

Symbolizing by individuals who feel incomplete does not require much effort expenditure or sacrifice to restore feelings of identity‐goal completeness. Thus, effortless slacktivist behaviors may still function as symbolic indicators of progress toward one's aspired‐to identity goal. Previous research has shown that even merely expressing intentions to act in line with one's identity goal can serve as a self‐symbolizing option when participants feel incomplete [16]. This aspect of self‐symbolizing is particularly interesting in light of recent findings regarding the use of social media when pursuing one's identity goals.

## 3 Easy but Effective Ways to Self‐Symbolize on Social Media

Recent experiments suggest that a sense of completeness regarding one's identity goal can be quickly achieved by posting relevant indicators on social networking sites. By sharing symbols of achievement online (e.g., a photograph wearing a white coat or court attire), individuals highly committed to an identity goal—such as becoming a good physician or a successful lawyer—may turn to platforms like Instagram to self‐symbolize. By doing so they can easily find an audience, register their symbols on it, validate the aspired‐to identity, and thus achieve a sense of completeness.

In a set of three experiments [17], it was found that highly committed medical and law students experience incompleteness (or completeness) in their professional identity goal and then provided access to Instagram. Specifically, students in the state of incompleteness were hypothesized to increase their online posting of indicators of goal attainment, such as photographs depicting identity‐related symbols (e.g., medical coats, court clothes). In Study 1, incomplete medical students posted more medicine‐related photographs on Instagram than their complete counterparts. In Study 2, law students experiencing incompleteness posted more law‐related photographs on Instagram, and even tended to write more law‐related captions and hashtags compared to participants who felt complete. This second study replicated Study 1's findings and clarified that compensatory online self‐symbolizing specifically relates to one's incomplete goal (law career) and not to other nonpertinent domains (university life). Finally, in Study 3, medical students feeling incomplete only engaged in online self‐symbolizing when their incompleteness referred to their career goal and not to other careers they did not aspire to (a law career). Taken together, all these studies demonstrate that a sense of incompleteness regarding one's career goal can be quickly compensated for on social media sites, by posting more indicators related to success regarding that special identity goal (e.g., hospital pictures, lawyer‐related hashtags).

A more process‐focused experiment [18] deepened the understanding of how compensatory online self‐symbolizing works. To test whether online posting of identity‐related symbols is able to resolve identity‐goal incompleteness and its effects, the authors experimentally varied the sense of incompleteness (vs. completeness) in a sample of committed medical students and then provided an opportunity to restore completeness through the posting of self‐symbolizing (vs. neutral) content on Instagram. Results showed that identity‐goal incompleteness induced a series of orienting effects—including impulsiveness in online posting, disinterest in others’ posted contents, feelings of irritation, and the narrowing of attention. Importantly, all of these orienting effects disappeared when students were provided and used the opportunity to post compensatory, self‐symbolizing content on Instagram (e.g., photographs framing medical coats and stethoscopes). These findings suggest that posting identity‐related symbols on social media sites can easily reestablish completeness.

In line with the symbolic self‐completion theory, moral identity can manifest itself in the commitment to pursue the identity goal of being a moral person. This aspired‐to goal is then shaped and defined by symbolic activities that individuals engage in to signal their goal commitment. A broad range of self‐symbolizing options exists, and social media offer an easily accessible—yet still effective—way to create a sense of completeness. Importantly, as in the case of other identity goals, this symbolic nature of moral identity striving, even when played out in low‐effort forms, can still have tangible societal and biospheric consequences. We will illustrate this by discussing proenvironmental moral identity goals and the phenomenon of moral slacktivism within this context.

## 4 Proenvironmentalism as a Morality Issue

Pursuing the goal of becoming a green person aligns with aspirations for being a moral person, as proenvironmentalism is firmly grounded in morality. For example, the moral foundations theory [19] identifies five evolutionary significant foundations to account for the diverse composition of people's moral value systems. Among these, care/harm and fairness/cheating are the individualizing foundations that are focusing on individual people. These foundations support proenvironmental actions by emphasizing concern for others’ well‐being and justice, values that align closely with environmental protection goals.

Another theory focusing on values, the norms‐activation theory [20] posits that different moral norms, personal norms, and values in particular, are driven by two main processes: awareness of consequences and ascription of responsibility. Accordingly, individuals take up altruistic behaviors when they realize that their decisions have consequences for the welfare of other people, and when they feel personally responsible for the social consequences of these decisions [21]. Altruism, as a value, thus lies at the roots of individuals’ conservation efforts, when individuals recognize what negative consequences unecological behaviors may have for others and feel responsible for their unecological actions [22].

Moreover, concern for the environment is also related to prosociality and fairness, inherently moral values. For example, proenvironmental attitude was found to be related to a higher chance of participants’ keeping their word in terms of showing up at a laboratory experiment on time [23] or playing fair in the common resource dilemma task paradigm [24]. Many proenvironmental behaviors such as energy conservation within groups can be seen as a common resource dilemma. Such decisions affect collective well‐being and individual actions have far‐reaching consequences for others [25, 26]. Because individuals juggle between short‐term selfish benefits and long‐term collective consequences for all members, these common decisions can be viewed as moral dilemmas as well [27].

Viewing morality through the lens of consequences for others is not the only way to establish its link to proenvironmentalism. The value–belief–norm theory [28] argues that people's concerns for environmental issues are shaped by their general values centered on self‐interest, the well‐being of others, and on the biosphere [29]. Caring for the environment can go beyond the awareness of consequences for themselves and others by focusing on an abstract valued entity such as nature. Holding the belief that nature is valuable in its own right translates into enhanced moral reasoning [30]. And moral considerations such as thoughts about fairness and justice predict attitudes focused on protecting the biosphere. This aspect of biospheric values differentiates proenvironmentalism from other moral domains—such as social or political—that primarily center on concern for others.

In sum, this short review of research on the link between environmentalism and morality suggests that moral concerns are inherently bound to proenvironmentalism. Norms of care, fairness, and justice as well as responsibility have been consistently identified as foundational to shape people's proenvironmental attitudes and behaviors. It seems justified therefore to target environmental concerns and their effects on respective behaviors when it comes to a better understanding of the pursuit of moral identity goals.

## 5 Proenvironmental Identity Goals

The identity goal of becoming a green person can manifest itself in self‐symbolizing actions such as signing an online petition, sharing a post about climate change, or adding a green badge to one's social media profile. According to the symbolic self‐completion theory, these symbols (indicators of goal progress) provide a sense of completeness regarding this very identity goal, thus reducing feelings of incompleteness. As identity‐goal striving is open‐ended, individuals may easily compensate for incompleteness by engaging in new symbolizing when their standing is questioned [31].

Some researchers analyzed [1] whether questioning/validating the purchase of green products intensifies/hampers subsequent green behaviors in people with the identity goal of being green. They found that negative feedback on purchasing green products led to more subsequent recycling compared to positive feedback, with participants who did not receive any feedback sitting in between. Moreover, participants who did receive positive feedback evinced a decreased cognitive accessibility of goal‐related constructs in a lexical‐decision task compared to participants who had received negative feedback, suggesting that feedback validating one's green purchasing choices is associated with heightened goal completeness.

Similarly, participants who had received positive feedback recalled a green patch as less green compared to negative feedback participants, suggesting that the urge to attain the identity goal of being green was weakened by positive as compared to negative feedback. This pattern of results was also observed in studies [32] showing that induced incompleteness regarding the identity goal of being an environmentalist led to stronger proenvironmental intentions than induced completeness.

Another relevant recent research paper [14] focused on vegans, as a key reason to convert to veganism is concern for the environment [33]. The two studies reported show, however, that even such a motivated group as vegans might succumb to temptations and prefer food that is nonecological, unsustainable, and transported from distant countries. Eco‐friendly vegans were presented with a menu of three dishes: a soup, a burger, and a cake with each dish having two options. One of the two options was always eco‐friendly, for example, a beet‐root soup with cumin or a burger with celery—products grown in Poland where the studies were conducted. The second option was always far from being eco‐friendly, but more appealing. For example, a sweet potato soup with coconut milk or a burger with avocado. After receiving the (in)completeness induction—that is either negative, positive, or neutral feedback on how vegan and eco‐friendly their everyday eating behaviors are—participants made three choices from their menu. Eco‐friendly vegans who experienced completeness were more likely to choose the nonecological options compared to those who experienced the state of self‐incompleteness. Moreover, in Study 2, it was found that individual differences such as self‐control as a trait and the need for pleasure did not moderate the negative behavioral effects of the states in which vegans felt complete. Apparently, the observed motivational drop associated with feelings of completeness and the motivational enhancement associated with incompleteness are universal and go beyond individual differences.

People who commit themselves to the identity goal of being a green person become persistent and loyal advocates for addressing climate change and promoting actions that protect the planet in a committed and sustained manner. Unlike a discrete task goal (e.g., to sort out one's waste in the office at the end of the day), the moral identity goal of being a green person who aspires to live a life that sustains our planet will lead individuals to continuously try to live up to this aspiration. One caveat of pursuing a long‐term, proenvironmental identity goal is that a sense of completion can be achieved through easy, symbolic actions. In the case of other identity goals—such as being a religious person or a feminine woman—the fact that symbolic actions may not be highly effective or instrumental is of less importance. In the case of proenvironmental actions, however, their effectiveness for the protection of the environment is an important issue.

## 6 Consequential Proenvironmental Behaviors

As natural resources deplete and climate change has become an increasingly urgent issue, simply voicing mottos such as “Let's wait and see!” is no longer sufficient [26]. Recently, researchers and policy makers heighten the importance of addressing consequential proenvironmental behaviors that have a measurable positive impact on the environment [34, 35]. Such an approach, however, has not always been a dominant one in the field. Initially, all proenvironmental behaviors, even those requiring little effort, such as voicing proenvironmental slogans or liking conservation posts online, were seen as valuable contributions to mitigating climate change. This view was supported by the idea that engagement in even effortless actions may lead to positive spillover effects: behavioral chain reactions that lead individuals from less to more demanding forms of engagement [36, 37, 38]. For example, voicing proenvironmental slogans on social media might translate into householders lowering the temperature when doing laundry.

Theorizing behind positive spillover effects is based on the assumption that performance of a single behavior triggers changes, which in turn may lead to engagement in further relevant, often more demanding actions [39]. Established theories such as the dissonance theory [40], the self‐perception theory [41], or more recently the Campbell paradigm [42] provide the groundwork for understanding the underlying mechanisms of this effect. For instance, dissonance theory explains spillover effects in terms of people's need for acting coherently as discrepancy in one's actions is seen as resulting in aversive tension. According to the self‐perception theory, people infer a tendency to act in one way or another from what they have been doing in the past. In line with this theorizing, it was found that labeling behaviors performed by participants as environmentally friendly led them to act in a more proecological manner on a subsequent occasion [43]. In another study supporting this reasoning, respondents’ declarations that they drove a vehicle in a cost‐effective manner correlated with the intention to reduce meat consumption a year later [44]. Finally, the Campbell paradigm offers a somewhat different explanation of the spillover phenomenon. This framework proposes that both easy (e.g., supporting proenvironmental issues on social media) and demanding proenvironmental behaviors (e.g., commuting by bike) are tangible manifestations of the same latent proenvironmental attitude, even if they are not strongly associated in terms of simple Pearson correlations [42]. Accordingly, spillover effects are seen as primarily driven by behavior‐based attitude change rather than by targeting a single behavior [45].

Even though spillover effects provided hope that already the slightest first steps toward nature conservation might lead to stronger actions down the road, recent meta‐analyses cast doubts on this optimism. For example, a comprehensive review [46] confirmed that positive spillover effects do occur, but the effect sizes are negligible. The effect of engagement in the first behavior on the intention to perform the second was found to be weak (*d* = 0.17), and on the performance of actual second behavior close to zero (*d* = −0.03). Moreover, positive spillover effects were more likely to be observed when both proenvironmental behaviors belonged to the same proenvironmental subdomain such as water saving or waste management. Similarly, other researchers [47] using a Bayesian meta‐analytic approach found strong evidence for an overall null effect of behavioral spillover.

Another problem is that behaviors differ in the degree to which they afford positive effects for the environment, that is, they differ in the extent to which they are consequential [48]. For example, deciding to commute by bike is more impactful than recycling, and recycling is more impactful than supporting proenvironmental issues on social media. These differences should affect the person's willingness to engage in these behaviors especially among individuals concerned about the environment. As a consequence, commuting by bike should be the most desirable action of the three and thus be more readily engaged in. But engaging in one of the many available proenvironmental behaviors is also affected by the person's respective self‐efficacy feelings: How certain is the person that the selected behavior can be performed successfully? Accordingly, whether people do engage in one or the other of these behaviors does not only depend on how impactful they are perceived in terms of proenvironmental consequences, but also whether the individual feels capable of executing these behaviors.

Whether certain behaviors are more or less environmentally consequential can be quantified in a standardized way through measures such as carbon and ecological footprints [49]. Despite advancements in the ways of measuring environmental impact, however, even individuals who are environmentally conscious often misjudge which actions are genuinely beneficial or harmful for the environment [50]. For example, people tend to overestimate the benefits of low‐impact behaviors such as recycling or avoiding plastic packaging and to underestimate the impact of dietary choices and modes of transportation [51, 52]. Given these findings, clearer public guidance and engagement in enacting impactful environmental behaviors qualifies as an important task for researchers and policy makers [53]. At the same time, the growing popularity of social media and virtual forms of environmental advocacy contributes to even more ambiguity in understanding of which proenvironmental actions are the most appropriate to support the cause. In the next section, we will therefore present both negative and positive aspects of symbolic manifesting people's proenvironmental identity goals on social media.

## 7 Proenvironmental Slacktivism on Social Media

Since social media sites allow the continuous sharing of identity‐related symbols, using these platforms for online activism may give individuals a premature feeling of completeness—before their intentions translate into impactful action. This, in turn, can reduce their urge to take further steps that effectively support or advance their cause. This should be particularly evident in those activists highly committed to describing themselves as sustainers of a specific cause such as protecting the environment. As a negative consequence, using social media to attract and mobilize people may have the unwanted effect of dispersing the energies of these active and dedicated supporters.

Moreover, since sharing opinions and intentions online qualifies as an always‐at‐hand, simple but effective shortcut to restoring completeness, highly committed activists that temporarily feel incomplete toward their identity goal could be drawn to engage in what is sometimes referred to as slacktivism (low‐effort engagement that may be ineffective, but can symbolize one's identity) instead of engaging in effortful, but more consequential actions when incompleteness is experienced. More specifically, the incomplete proenvironmental person might prefer a rapid posting of relevant symbols instead of mobilizing additional efforts or waiting for the right occasion to engage in a more consequential proenvironmental action. Also unfortunate, a like‐minded audience will be preferred, since being confronted with people who speak up would not effectively achieve the more immediate goal of a person in a state of incompleteness—reestablishing completeness as fast as possible (see the research on echo‐chambers [54]). Finally, self‐symbolizing efforts are known to be especially effective when registered by others, as the possession of indicators of completeness will thus become a social reality. Because identity goals are typically socially defined, the effectiveness of indicators of goal completeness (identity symbols) depends on the mere being noticed—not being positively evaluated—by others [9, 12], which can easily be achieved via online posting.

No wonder then that online activism being characterized by low‐cost, low‐risk actions such as signing petitions or changing profile pictures has been referred to as slacktivism. It may contribute to the awareness of social problems, but is rightfully criticized for being societally ineffective as it primarily serves to enhance participants’ feel‐good factor [55]. Slacktivism can symbolize moral standing by demonstrating support for social causes, but it may also lead to reduced readiness for more substantial activism such as engagement in consequential proenvironmental behaviors, in particular [56]. A striking example of this phenomenon is a field study [57], in which an online campaign asking for donations reached 6.4 million users, who liked or shared the campaign, but only 30 donations in total were made—symbolizing a green identity online did not translate into tangible action.

From a different perspective, however, it is important to consider that even when online activism may lead to slacktivism, it may still have some positive consequences. For example, the possibility to engage in low‐effort online actions in support of a social cause—such as changing one's profile picture to a “green” image, reposting a blog, or simply expressing one's opinion on environmental issues—may encourage participation from less committed individuals. These less engaged individuals may be willing to sign petitions, donate small amounts of money, or share environmental guidelines. Even when not driven by the noblest intentions targeting the most consequential actions, then, they are nonetheless contributing to sustaining the environmental cause [58, 59, 60].

More importantly, slacktivism can also help disseminate messages about environmentalism, amplify their public resonance, and potentially foster a broader cultural shift around the issue. The ephemeral and lightweight nature of the content typically shared on social networking sites like Instagram predisposes individuals to use these platforms for quick acts of self‐symbolizing, and by doing so, to promote the rapid and widespread circulation of emerging, new ways of life. This allows the easy spreading of messages about environmentalism, for instance, that reach broader audiences and resonate more easily across different social groups [61].

One of the most interesting positive aspects of slacktivism, in our view, is that it may help committed individuals remain engaged in pursuing their identity goals when facing incompleteness. As implied by the symbolic self‐completion theory [9], when individuals experience failures or any other situation suggesting that they are falling short in their identity goal, they start looking for alternative symbols of completeness to compensate. In such cases, having an easily accessible way to reestablish completeness—even if merely symbolic and temporary, such as posting a “green” profile picture and reminding their followers of past successes—can prevent genuinely committed activists from giving up in the face of difficulties (e.g., an eco‐friendly person that feels their persuasive efforts are not making a real difference regarding their family members’ habits). Later, the committed person who has overcome feelings of inadequacy by quick and easy self‐symbolizing on social media can return to reflective deliberation on how to invest renewed energy in striving toward their identity goal by more effortful consequential self‐symbolizing.

## 8 Potentially Helpful Self‐Regulation Strategies

As a state of completeness regarding identity goals can be acquired in a relatively quick and easy way via online posting, the question arises what self‐regulation strategies individuals can adopt to escape this trap and take effortful action to promote their moral identity goals. A recent line of research [62] investigated whether inducing a deliberation mindset in incomplete individuals by offering a choice between different options to engage in compensatory self‐symbolizing will make them prefer the more effective ones (i.e., effective in terms of successful actual goal pursuit) and not just jump on any available mere symbolic option.

The authors found that incomplete individuals who were put into a deliberation mindset [63] did prefer the more consequential options, even when these options were associated with higher effort expenditure. These findings suggest that incomplete individuals can protect themselves from falling for quick and easy online self‐symbolizing when they mentally prepare themselves for coping with potentially upcoming experiences of incompleteness, prospectively lining out the many different ways they could use to engage in compensatory self‐symbolizing of completeness. The deliberation mindset is relatively easy to induce, so in practice, giving participants a choice between two proenvironmental options might increase the likelihood that they will go for actions that are more effective and consequential for the planet.

A completely different but equally effective self‐regulation strategy might be committing oneself to a selected effective way of coping with an anticipated experience of incompleteness. This can be done by making prospective if‐then plans [64, 65]. The self‐regulation strategy of if‐then planning is very simple but highly effective in automating the initiation of a desired critical behavior. One only has to specify the anticipated critical situation (*x*) in the if‐part of the plan, and an effective coping response (*y*) in the then‐part of the plan and then connect the two by stating “If *x* occurs, then I will enact *y*!” For instance: “If I start worrying whether I will ever become a green person, then I will tell myself: Hop on your bike and drive to the market to shop local products!”

Such if‐then planning can be combined with the self‐regulation strategy of mental contrasting [66] that helps people to more clearly detect what they want to achieve and what kind of obstacles they need to overcome in order to move forward. The combined self‐regulation strategy is called MCII (i.e., mental contrasting with implementation intentions, also referred to as WOOP [48, 67]). But the mere self‐regulation via mental contrasting alone is found to be already effective on its own via heightening people goal commitments. More strongly committing oneself to the goal of becoming a green person via engaging in mental contrasting is thus also an effective route to go as high commitments to identity goals help people fight back when incompleteness—the feeling that one has failed to act on one's identity goal—is experienced.

Finally, beyond self‐regulation, it should be acknowledged that even environmentally concerned individuals are often unsure about which behaviors are more and which are less consequential for the environment. But broad, large‐scale informational campaigns may not always be the most effective educational approach, as individuals can become overwhelmed when presented with too many options [48]. One potential solution could involve implementing nudges, such as labels that clearly indicate the environmental footprint of a given product. These could function similarly to nutrition facts labels, providing consumers with accessible and comparable information. Additionally, real‐time feedback systems—such as displays indicating the environmental consequences of water or energy use—could help individuals make more informed environment‐protective choices [68].

## 9 Situating Identity‐Goal Perspective in the Existing Moral Identity Research

While the identity‐goal perspective is distinct from existing moral approaches, it shares some underlying assumptions. In the following paragraphs we will outline the main similarities and differences. In the moral psychology literature, three approaches to studying moral identityare most prominent: The *self model* [69] is influenced by rationalist moral philosophy, particularly in its emphasis on the centrality of moral reasoning. The *social cognitive theory* [70, 71] points out that moral identity operates as a central self‐schema. And finally, *moral self‐regulation* research examines how moral identity and prior moral (or immoral) actions influence future ethical behavior [72, 73].

In the self model, moral identity is conceptualized as an important integrated aspect of the self, emphasizing the importance of conscious moral reasoning and the pursuit of self‐consistency [69]. In this framework, moral motivation arises from the need to maintain self‐integrity, while willpower and self‐regulation capacity are needed to bridge the gap between moral judgments and moral action. Moreover, moral norms are seen as universally prescriptive—morality is not about subjective preferences (Who do I want to be?) but about standards that ought to apply to everyone (Who should I be?). In contrast, SCT does not conceive of moral identity as a fixed entity but as a dynamic, self‐defining goal that is actively pursued through ongoing behavioral and symbolic efforts while the individual moves between states of identity‐goal incompleteness and completeness [2].

Social cognitive theory offers a distinct and influential account of moral identity, emphasizing the role of learned cognitive structures and situational activation [7, 70, 71]. In the social cognitive theory, moral identity is conceptualized as a central self‐schema: an organized, context‐sensitive network of moral values, traits, and behavioral scripts shaped by personal experience and social modeling [5, 8]. Unlike the trait‐like stability that is assumed in rationalist models such as the self model, social cognitive theory emphasizes the malleability and dynamic activation of moral identity based on current situational cues and the *working self‐concept* [74]. Through processes of associative and observational learning, individuals internalize moral norms and values from significant others, institutions, and cultural contexts, modifying their self‐schemas across the lifespan.

The social cognitive perspective explicitly acknowledges both controlled and automatic processes in moral regulation [75, 76]. While conscious reflection can activate moral identity and guide behavior, much of everyday morality operates automatically through well‐practiced scripts and intuitive responses stored within self‐schemata [77]. Central to this account is the idea that not all self‐schemata are equally important or chronically active; instead, their influence fluctuates according to context, personal salience, and social validation. However, social cogntive theory is less explicit than self‐completion theory in formalizing the motivational forces underlying moral behavior. While empirical research highlights individual differences in moral identity centrality and the salience of moral issues has been identified as an important situational factor, the social cognitive theory does not systematically account for phenomena such as moral licensing or compensation that arise from motivational consequences following moral successes or failures [78]. In contrast, self‐completion theory addresses this gap by explaining fluctuations in moral motivation as the result of goal pursuit dynamics and the individual's ongoing management of perceived identity in/completeness [9].

Moral identity, whether conceptualized as a self‐concept, schema, or goal, serves as a powerful motivator of self‐regulating one's moral behavior [8, 69, 79], and a substantial body of evidence confirms that an activated moral identity often promotes greater consistency in moral actions [80]. Moral identities, such as being environmentally conscious, may differ from nonmoral identities (e.g., professional) in their sense of obligation [5]. While individuals may feel less compelled to act on nonmoral identities, moral identities often involve internalized norms that create a persistent urge to act in line with one's values [69]. However, there is some evidence

Showing that the influence of moral identity on behaviors is not always that straightforward. It is found that past moral or immoral behaviors also shape subsequent conduct through processes of self‐regulation [72, 78, 81]. That is, positive moral feedback or moral actions can lead to a relaxation of standards, a phenomenon known as moral licensing, while negative feedback or perceived transgressions can spur compensatory behaviors to restore one's moral self [82, 83]. While these findings of moral self‐regulation are not fully explained by the self model or the social cognitive theory, they do match the predictions of the identity‐goal perspective.

## 10 Conclusion and Outlook

The present paper offers a new perspective on morality by focusing on the moral identity goal of being a green person. With this example, we discussed relevant determinants, consequences, and the underlying processes behind green behaviors, including environmental slacktivism. Still, there is a host of open questions which we hope future research will address.

First, individuals are committed to a different degree to the various identity goals they entertain. Commitment determines the energizing power of an identity goal. Moral identity can thus be expected to be more influential for individuals who are strongly committed to being a moral person. Variations in commitment may help to explain inconsistencies in moral behavior research. For instance, some researchers find moral consistency effects, where small moral acts predict further ethical behavior, while others report moral compensation effects, including moral licensing [84]. One possible mechanism is that strong commitments to a moral identity‐goal buffer against licensing effects, reducing the tendency to disengage after symbolic moral actions [85].

Second, symbolic self‐completion theory [9] predicts that identity goals regulate behavior in systematic ways. Their energizing pull is the strongest when individuals experience moral identity incompleteness—a state that prompts efforts to acquire symbols of morality. In contrast, completeness leads to moral relaxation, as shown in moral compensation research [86]. This dynamic depends on commitment: only those strongly committed to a moral identity experience the tension that drives compensatory action. Empirical support comes from research [87] that induced moral identity incompleteness by prompting participants to recall past immoral actions. In response, individuals with high moral identity centrality felt greater guilt and were more likely to volunteer. Similar effects were found after lying in economic games [88]; highly moral‐identified participants were more honest and charitable. A key prediction of self‐completion theory is that symbols of morality are interchangeable. Acquiring any moral symbol—whether recalling past moral deeds, acting morally, refraining from wrongdoing, or even planning future moral acts—can create a sense of completeness, licensing future moral disengagement [81].

Third, identity goals can be thought of as organized knowledge structures [89], but in contrast to mere self‐schemata, identity goals have a self‐regulatory function that focuses on goal progress. Importantly, the different content elements of identity goals are associatively connected. Thus, encountering environmental cues encoded in the various goal contents can make the green identity goal more accessible [77, 90, 91]. In turn, holding a goal active increases the accessibility of its content. However, the cognitive principles of frequency and recency of activation that one expects to increase the accessibility of associated content should be overridden by motivational principles of goal activation: once a goal is completed, accessibility of the goal content decreases.

Thinking about identity goals as organized knowledge structures means that a person may strive to realize many self‐defining goals at the same time. For example, one might aim to become a good professional, to enjoy hedonic pleasures, and to maintain a healthy athletic lifestyle at the same time. Past research has shown that one goal may override another. For example, individuals who were experimentally induced to experience incompleteness regarding their professional identity goals were more likely (compared to an identity‐goal complete control group) to compensate by endorsing immoral self‐symbols, but only if they were committed to the professional identity goal [92]. Thus, the likelihood of engaging in green behavior depends not only on commitment and completeness versus incompleteness states of the green identity goal, but also on other currently active goals as well. For instance, riding a bike to work on a rainy day may align with one's environmental identity but conflict with his or her hedonic identity goal. In other words, riding a bike may be highly instrumental regarding an environmental identity goal, but counterfinal regarding a hedonic goal. But multiple identity goals do not have to stay in conflict as for instance slacktivism may symbolize both green and professional identities or biking, recycling and slacktivism may all symbolize a green identity goal. Future research could, for example, explore how multifinality (a single behavior leading to symbolizing many identity goals) might make slacktivism more appealing. For instance, sharing a proenvironmental post on social media could simultaneously: Symbolize one's green identity (self‐completion), signal professional engagement (if the cause aligns with one's career), and fulfill hedonic goals (e.g., social validation). Understanding such goal configurations could clarify why some people prefer slacktivism over more effortful actions.

Related recent studies found that incompleteness may spread between identity goals that are associated via similar reasons for their pursuit. For instance, similar reasons for goal pursuits were leading identity‐goal incomplete people to engage in self‐symbolizing in overlapping goal domains when no symbols to compensate for the incomplete goal were readily available [15, 93]. Reasons along which identity goals may overlap can be the moral values associated with these goals. Identity goal overlap in terms of morality should be related to moral identity complexity [39]. Moral identity is not a uniform construct; rather, an individual's moral concerns can differ across various social roles (e.g., as a coworker, friend, or family member). Moreover, if multiple identity goals are active simultaneously, people might prefer activities that can symbolize all of their active goals at the same time [79]. Future studies should explore whether people holding both identity goals of being a vegetarian (for animal welfare reasons) and of being environmentally friendly, might prefer a vegan but non–eco‐friendly lunch option when their vegetarian identity goal is incomplete but their proenvironmental identity goal is complete. However, when both identity goals are incomplete, they might take the effort to find a lunch option that satisfies the demands of both of them.

Fourth, the discussion of slacktivism in this paper has largely focused on its direct environmental ineffectiveness. Yet, online proenvironmental behaviors may also carry indirect positive effects, for instance, by influencing the attitudes and behaviors of others in one's social networks [94]. Whether individuals who engage in slacktivism are aware of or motivated by this potential for indirect impact remains an open empirical question. Do people who share a proenvironmental post believe they are contributing to meaningful change by inspiring others? Or are they primarily driven by the identity‐related goal of feeling complete as an environmentally responsible person, largely indifferent to actual environmental outcomes? Future studies could use experimental designs to show what motives stand behind slacktivism and what the role of social context and social exposure is. Understanding whether slacktivists perceive themselves as catalysts for broader social change, or merely as symbolic actors, would have important implications for communication strategies aimed at converting online engagement into sustained, impactful action.

## Author Contributions

Katarzyna Byrka, Simona Sciara, Johannes T. Doerflinger, and Peter M. Gollwitzer conceptualized the topic, contributed original sections, and provided substantive feedback to each other to enhance clarity and scope.

## Conflicts of Interest

The authors declare no conflicts of interest.
